# Some Thoughts on a City’s Milk Supply

**Published:** 1902-06

**Authors:** 


### SOME THOUGHTS ON A CITY'S MILK-SUPPLY.

THE science of dairying is at present making rapid strides. This subject is being developed and taught in dairy schools connected with agricultural colleges and State universities, and in numerous bacteriological and chemical laboratories at home and abroad.

Very much more is now known of the relation of foods, management of cattle, health of cows, sanitary and unsanitary stabling conditions, methods of milking, and the treatment of milk after it is drawn and while en route to the consumer, than ever before. Further information in respect to all of these and other related dairy questions of vast economic and sanitary importance is being rapidly gathered and made available for the use and benefit of producers, distributers, and consumers of milk and other dairy products.

These questions are scientific, and are being developed along scientific lines by men especially trained for this work. All of these developments have important bearings on the milk-supply of cities, and should be applied so that all branches of the milk trade and consumers of milk shall be benefited.

Years ago milk venders were permitted to sell as milk anything that had the general appearance of milk and tasted like milk. It mattered not whether this fluid was robbed of its cream, diluted with water, charged with filth, visible and invisible, or obtained from diseased animals fed on putrid food and kept under the worst imaginable conditions. To check fraud and, in a certain measure, to protect the public from disease, laws have been enacted under which cities and towns have appointed milk inspectors. These officials are required to see that milk sold within the limits of their jurisdiction does not fall below a certain chemical standard established by law. In this way the public is protected against gross adulteration, and is not required to pay more for milk than it is worth as gauged by the chemist's analysis.

Other requirements have since been established that have led to the abolition of some of the most flagrant and openly disgusting conditions under which milk was formerly produced. While the large city "swill milk" cow stables are a thing of the past, other objectionable and dangerous conditions and practices exist that are harmful to the consumer and injure the milk trade.

For the past ten years or more the milk-inspection service of Philadelphia has consisted in the enforcement of a legal standard for milk, which requires that it shall contain a certain percentage of fat and other solids, thus protecting the pocket of the purchaser. The authorities charged with the inspection of milk in Philadelphia have recognized and have shown in their reports and by their action their appreciation of the high importance of the sanitary conditions under which milk is produced and handled. But when attempts have been made to place in operation measures intended to improve milk from the sanitary side these attempts have been so sweeping and arbitrary that they have served to restrict the usefulness of the work of the milk-inspection division. As an instance, the regulation in reference to milk from Chester County may be cited: It came to the attention of the chief of the milk-inspection division that tuberculosis prevailed among certain herds in Chester County, and in utter disregard of the fact that this was not a new thing, that the disease had prevailed there for many years, and was quite as prevalent among cattle in all other sections within the same distance of Philadelphia, an order was issued to the effect that milk from Chester County should not be received and its sale should be prohibited in Philadelphia. It is needless to say that this order created an immense disturbance, was naturally barren of beneficial results, and it was soon withdrawn. Its only effect was to bring the milk-inspection division into disrepute, for it exposed its ignorance of the producers and of the industry it is called upon to oversee. As another instance of similar character, attention may be called to the order that was issued in relation to the testing of cows producing milk for sale in Philadelphia. This order emanated from the Board of Health, and provided that all cattle supplying milk for sale in Philadelphia should be tested with tuberculin, and only the milk from non-reacting cows should be offered for sale. Dealers were, by this order, required to obtain certificates from their producers, showing that the cattle were examined in accordance with this regulation. There was neither law nor machinery for enforcing this order. It was intensely unpopular among the producers, who would have suffered incalculable loss if it had been enforced. At that time there was not tuberculin enough in the United States to test a tenth of the cows covered by this order, and that Philadelphia did not suffer a milk famine and the milk trade become utterly demoralized was due only to the fact that no effort was made to enforce this impossible regulation.

The prohibition of the sale of skim milk also illustrates an incomprehensible attitude and peculiar conception of the duties of the milk-inspection division. Skim milk is a very cheap, nutritious, wholesome, and useful food. It sells for a price that brings it within the reach of people who cannot afford to buy whole milk and who are deprived of the advantages of a partial milk diet if the sale of skim milk is prohibited.

The city is to be congratulated upon the fact that its present Executive at once recognized this fact, and was inflexible in his position that the poor man's family should not be deprived of an important, wholesome, and unobjectionable addition to their meagre diet.

The improvement of the milk-supply of Philadelphia has, it is believed, been hindered in some instances by the harassing surveillance to which the earnest advocates of improved systems have been subjected.

The attitude of the milk-inspection office in relation to the sale of milk in bottles, which is generally recognized as a great advance in sanitary distribution, was reactionary and obstructive.

Attempts of the Philadelphia authorities to secure changes in the State laws relating to the milk inspection have uniformly during the past ten years resulted in failure. The changes that were suggested by the milk-inspection division were actively opposed by the milk producers and milk dealers, and did not receive the support of the general public.

The office of the milk inspector should be in touch with all portions of the milk trade, and should be in sympathy with every attempt to improve the milk-supply of the city. The chief milk inspector should be thoroughly conversant with the conditions under which milk is produced and the problems that confront the producer and shipper. He should be informed as to the transportation, protection, and distribution of milk. He should know the influence of different sanitary conditions, diseases, and foods on the quality and wholesomeness of milk. He should keep pace with the modern developments of dairy science, and should see to it that the useful and practical discoveries following each other with such rapidity are applied for the protection and benefit of milk consumers and the milk trade.

The chief milk inspector should not only recognize that milk is dangerous under some conditions, but he should be able to avoid these dangers. He should not attempt to minimize the harm that may result from the consumption of unwholesome milk by placing all milk in disrepute and by endeavoring to check the sale and use of this product. On the contrary, he should know that milk is one of the most wholesome and nourishing articles of diet, and that at its present price there is scarcely another food to compete with it on the score of economy, and while its consumers should be protected, the use of milk should also be encouraged.

In other words, the chief inspector of milk should have complete knowledge as to how milk is at present produced, cared for, and distributed. He should also know the best methods of producing, protecting, and distributing milk, the extent to which it is possible to improve existing conditions, and the means to be adopted to this end.

The office of the chief milk inspector should not lack the sympathy and support of all branches of the milk trade.

It is our opinion, after mature deliberation, that the milk-supply of this city could be materially improved and the consumption of this food increased without further legislation or expense to the city, if harmony and a better understanding existed between the producers and distributors on one hand and a thoroughly equipped chief milk inspector on the other.

